# Death of Dr. Edmunds—Action of the Erie Co. Medical Society

**Published:** 1869-09

**Authors:** 


### Editorial Department.

### Death of Dr. Edmunds—Action of the Erie Co. Medical Society.

At a meeting of the Erie County Medical Society, held on the evening of the 27th ult., called to take action on the occasion of the death of Dr. J. J. Edmunds, the Vice-President, Dr. Miner, on taking the chair, spoke as follows :

GENTLEMEN: I am again called upon to announce the death of one of the members of this Society, Dr. James J. Edmunds, who died, after a long and painful illness, August 25th, aged fifty years. Dr. Edmunds was a graduate of the Buffalo Medical College in 1851, and has practiced his profession in Buffalo since that time. He has long been a worthy member of the Erie County Medical Society and of the Buffalo Medical Association. He was elected Coroner of Erie County in 1863; was President of the Board of Health, and held at various times many offices of honor and trust. Dr. Edmunds was an honest, earnest worker in his profession, though constantly struggling with embarrassments and misfortunes—disease always casting shadows over his professional hopes and expectations. He had a modest, unassuming, gentle spirit, and his real merits were largely unappreciated except by the few who knew him best. He battled manfully with his woes, and is at rest. He leaves a wife, three sons and a large circle of friends to mourn his early death. You will please take such action as you deem appropriate upon such an occasion.

Dr. Eastman also spoke in kind and complimentary terms of the deceased, and closed by moving the appointment of a committee of three to draft resolutions expressive of the sense of the society on the occasion. The motion was adopted, and Drs. Eastman, Johnson and Barnes, Jr., were appointed such committee, and subsequently presented the following resolutions :

*Resolved,* That in the death of Dr. James J. Edmunds, we mourn the departure of a worthy brother and friend, who has ever exhibited noble qualities and energy in the practice of our profession. Honest and simple in his nature and habits, open and frank in the expression of his sentiments, manly, generous and just in his professional intercourse, he has endeared himself to our kind and grateful remembrance.

*Resolved*, That we will attend his funeral on the 29th inst., and wear the usual badge of mourning.

*Resolved*, That a copy of these proceedings be transmitted to the family of the deceased, and that the same be published in the Buffalo *Medical Journal* and in the city papers. Adopted.

